# Prevalence and influencing factors of malnutrition in maintenance hemodialysis patients in China: a systematic review

**DOI:** 10.3389/fpubh.2026.1771997

**Published:** 2026-03-06

**Authors:** Yanan Ban, Hailin Zhang, Qianqian Wei, Fei Chen, Xiaoyan Wen, Lixia Yin

**Affiliations:** 1 Blood Purification Centre, Lianyungang Clinical College of Nanjing Medical University, Lianyungang, China; 2 Department of Nursing, Lianyungang Clinical College of Nanjing Medical University, Lianyungang, China; 3 Department of Nursing, The Fourth People’s Hospital of Lianyungang, Lianyungang, China; 4 Blood Purification Centre, The Affiliated Lianyungang Hospital of Xuzhou Medical University, Lianyungang, China

**Keywords:** influencing factors, maintenance hemodialysis, malnutrition, meta-analysis, prevalence

## Abstract

**Background:**

The number of patients with chronic kidney disease (CKD) continues to rise in China, where maintenance hemodialysis (MHD) is a primary treatment. However, long-term hemodialysis is frequently associated with malnutrition, which severely compromises patients’ quality of life and prognosis.

**Objective:**

To systematically evaluate the incidence and influencing factors of malnutrition in MHD patients by using Meta-analysis methods, thereby providing a basis for early clinical identification and intervention.

**Methods:**

We systematically searched PubMed, Web of Science, the Cochrane Library, CINAHL, China National Knowledge Infrastructure (CNKI), VIP, Wan Fang Data and SinoMed. The search covered the period from database inception to October 10, 2025, for studies investigating factors influencing malnutrition in MHD patients. Data were analyzed using Stata 15.0. A random-effects model was applied in cases of substantial heterogeneity (*I*^2^ ≥ 50%), otherwise a fixed-effects model was used. Publication bias was assessed using Egger’s test, and the trim-and-fill method was employed if necessary.

**Results:**

A total of 26 studies involving 5,055 patients were included, and the overall incidence of malnutrition in MHD patients was 46.9% (95% CI: 41.8–52%). Meta-analysis showed that age (OR = 1.509), body mass index (BMI) (OR = 1.544), dialysis vintage (OR = 2.265), urea clearance index (Kt/V) (OR = 2.019), serum C-reactive protein (CRP) (OR = 3.013), high-sensitivity C-reactive protein (hs-CRP) (OR = 2.104), protein intake (OR = 3.018), frequency of dialysis (OR = 2.100), depression (OR = 2.671), anxiety (OR = 2.531), monthly household income (OR = 1.563), serum albumin (ALB) (OR = 1.115), frequency of erythropoietin (EPO) use (OR = 1.506) and duration of dialysis per session (OR = 1.879) were the main influencing factors (*p* < 0.05).

**Conclusion:**

Malnutrition is present in 46.9% of maintenance hemodialysis patients. Individualized assessment and intervention targeting these key factors are essential to improve nutritional status and patient prognosis.

**Systematic review registration:**

https://www.crd.york.ac.uk/PROSPERO/, registration no. CRD42024596946.

## 1 Introduction

Chronic kidney disease (CKD) is widely recognized as a major global health challenge and has garnered considerable international concern (1). It is characterized by a progressive decline in renal function and structural damage in the kidney. As CKD advances to End-stage renal disease (ESRD), patients typically require renal replacement therapy (RRT), with hemodialysis (HD) being the most commonly used modality. However, patients undergoing long-term maintenance hemodialysis (MHD) are prone to malnutrition. This condition arises from various factors, including the loss of nutrients and trace elements, insufficient protein intake, inadequate dialysis, and a chronic inflammatory state. Additionally, the economic burden of treatment can lead to anxiety and depression, further exacerbating the risk (2, 3). Malnutrition is one of the most common complications in MHD patients and has been shown to significantly increase the risk of infections, falls, adverse cardiovascular events, and mortality. Moreover, it severely impacts patients’ quality of life and compromises treatment efficacy, with the severity of malnutrition correlating positively with mortality rise (4, 5). Therefore, understanding the incidence of malnutrition and related influencing factors in MHD population in China is of great significance for promoting the prevention and treatment of malnutrition. In recent years, with the popularization of maintenance hemodialysis technology and the extension of patient survival, the quality of life of MHD patients and the management of long-term complications have attracted more and more attention, among which nutrition issues have become a research hotspot (6). Early studies mainly focused on describing the epidemiological characteristics of malnutrition, and gradually developed to more in-depth explore its multi-dimensional influencing factors, the interaction with inflammatory state and psychological factors (7, 8), and the effect of personalized nutritional intervention strategies (9, 10). Although numerous studies on this topic have been conducted internationally, due to the differences in research methods, sample selection and regional differences, the results of existing studies are quite heterogeneous, and there is still a lack of systematic studies on the Chinese population. Therefore, this study aimed to systematically review the incidence of malnutrition and its main influencing factors among MHD patients in China through a meta-analysis, summarize the main influencing factors, and provide a scientific basis for early identification, intervention and management of MHD patients in clinical practice, so as to provide support for improving the prognosis and quality of life of patients.

## 2 Methods

### 2.1 Search strategy

We systematically searched the following electronic databases: PubMed, Web of Science, the Cochrane Library, CINAHL, China National Knowledge Infrastructure (CNKI), VIP, Wan Fang Data and SinoMed. Medical Subject Headings (MeSH) and free keywords were employed, covering terms related to dialysis, malnutrition, and influencing factors. The search period spanned from database inception to October 10, 2025. The detailed search strategy for PubMed is provided as an example in Table 1.

**Table 1:** Search strategies of PubMed databases

**Steps**	**Retrieval formula**
1#	Renal dialysis [Mesh]
2#	(Dialyses, Renal [Title/Abstract] OR Dialysis, Renal [Title/Abstract] OR Hemodialysis [Title/Abstract] OR Dialysis, Extracorporeal [Title/Abstract] OR Extracorporeal Dialysis [Title/Abstract])
3#	1# OR 2#
4#	Malnutrition [Mesh]
5#	(Malnourishment [Title/Abstract] OR Undernutrition [Title/Abstract] OR Nutritional Deficiency [Title/Abstract] OR Nutritional Deficiencies [Title/Abstract] OR Malnutrition [Title/Abstract])
6#	4# OR 5#
7#	Risk factors [Mesh]
8#	(Influencing factors [Title/Abstract] OR Determinants [Title/Abstract] OR Associated factors [Title/Abstract])
9#	7# OR 8#
10#	3# AND 6# AND 9#

### 2.2 Inclusion and exclusion criteria

Inclusion criteria were: (1) studies investigating malnutrition and its influencing factors in MHD patients; (2) cross-sectional or case–control study designs; (3) publication in either Chinese or English. Exclusion criteria included: (1) translated publications; (2) conference abstracts, guidelines, reviews, commentaries, or non-peer-reviewed materials; (3) studies rated as low quality or below in our methodological assessment.

### 2.3 Literature screening and data extraction

Two independent researchers screened for potential inclusion and extracted data according to predefined inclusion and exclusion criteria. If differences of opinion occurred during the screening process, third-party researchers were used to mediate and reach consensus. During data extraction, a prespecified data extraction form was used to collect and record the following key information: author, year, study region, sample size, prevalence of malnutrition, study type, assessment tools, influencing factors, quality evaluation tool and quality evaluation score. In case of missing data or unclear presentation, we have contacted the original authors for additional information. If the complete data could not be obtained, the article would be excluded.

### 2.4 Literature quality evaluation

The methodological quality of cross-sectional studies was assessed using the 11-item scale developed by the Agency for Healthcare Research and Quality (AHRQ) (11). Each item was scored as “yes” (1 point), “no,” or “unclear” (both 0 points), yielding a total score ranging from 0 to 11. Studies were then categorized as low (0–3 points), moderate (4–7 points), or high (≥8 points) quality. For case–control studies, quality was evaluated with the Newcastle-Ottawa Scale (NOS) (12), which comprises 8 items across three domains, with a maximum score of 9. Based on the NOS, studies scoring 1–3, 4–6, and 7–9 were rated as low, moderate, and high quality, respectively.

### 2.5 Statistical analysis

All data were analyzed by Stata 15.0 software. First, the heterogeneity test was performed, and the heterogeneity between studies was evaluated by the *I*^2^ statistic. When *I*^2^ > 50% and *p* < 0.1, it indicated that there was substantial heterogeneity, and the random-effects model was used for analysis. Otherwise, fixed-effects model was used for the meta-analysis. For studies with significant heterogeneity, subgroup analyses were performed to explore the source of heterogeneity. Simultaneously, sensitivity analysis was performed to verify the robustness of the analysis results, and Egger’s test was used to assess publication bias. A *p*-value < 0.05 was considered statistically significant.

## 3 Results

### 3.1 Results of literature search

**Figure 1:**
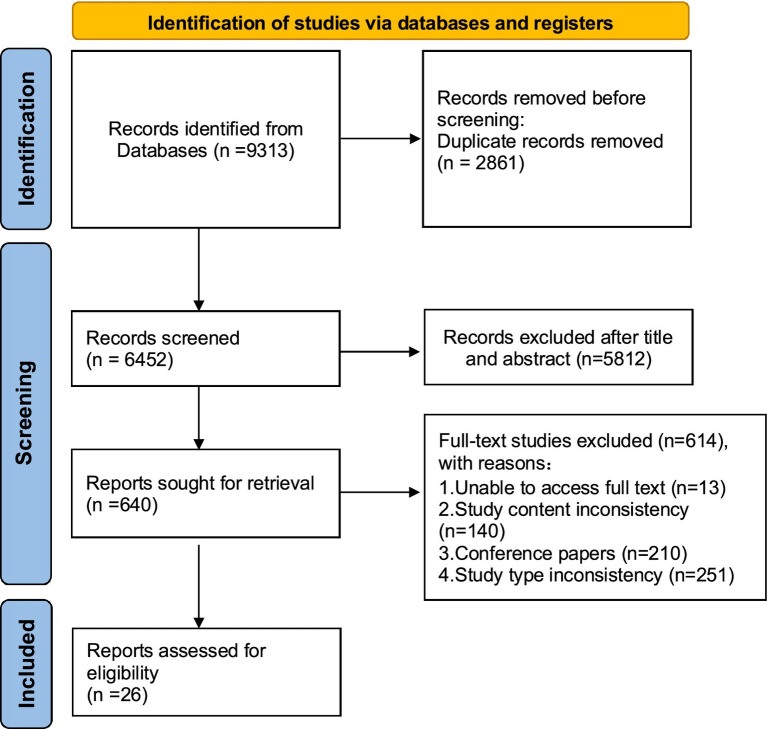
Flow diagram of the study screening process.

A total of 9,313 studies were retrieved, and 26 studies were included. The detailed study screening process is shown in Figure 1 and Table 1.

### 3.2 Basic information and quality evaluation of included studies

A total of 26 studies were included in this study (13–38), including 13 case–control studies and 13 cross-sectional studies, with a total sample of 5,055 cases, including 2,917 cases identified as well-nourished and 2,138 cases with malnutrition. Among the 26 articles, 20 were of medium quality and 6 were of high quality. The quality assessment results are presented in Supplementary Table 1. The basic information and quality evaluation results of the included literature are summarized in Table 2.

**Table 2:** Basic characteristics and evaluation results of included literature

**Author, year**	**Study region**	**Sample Size**	**Prevalence of malnutrition (%)**	**Study type**	**Assessment tool**	**Influencing factors**	**Quality evaluation tool**	**Quality evaluation Score**
Hu et al. 2023 (13)	Hanan	94	48.94	case-control study	MQSGA	1, 3, 4	NOS	7
Chen et al. 2024 (14)	Fujian	75	53.33	case-control study	MQSGA	3, 4, 5, 6	NOS	7
Han et al. 2022 (15)	Henan	118	55.93	case-control study	MQSGA	1, 3, 4, 19	NOS	6
Shi et al. 2016 (16)	Shanghai	120	58.33	case-control study	MQSGA	1, 3, 4, 19	NOS	6
Wang et al. 2 012 (17)	Beijing	125	48.80	cross-sectional study	SGA	4, 7, 11	AHRQ	5
Li et al. 2023 (18)	Beijing	147	40.14	case-control study	MIS	1, 20, 21	NOS	5
Gong et al. 2025 (19)	Shandong	120	35.83	cross-sectional study	MQSGA	3, 29	AHRQ	4
Qiu et al. 2022 (20)	Jiangsu	1228	42.02	cross-sectional study	MQSGA	1, 3, 4, 14	AHRQ	4
Xiao et al. 2017 (21)	Hunan	103	42.72	cross-sectional study	MQSGA	1, 6, 11	AHRQ	4
Weng et al. 2018 (22)	Jiangsu	200	37.50	cross-sectional study	MQSGA	3, 10, 13, 14, 15, 16, 17, 18	AHRQ	4
Chen H, 2020 (23)	Sichuan	88	69.32	cross-sectional study	Q-SGA	1, 2, 3, 4, 8, 26	AHRQ	5
Han et al. 2023 (24)	Henan	195	41.54	cross-sectional study	SGA	1, 3, 8, 11, 14	AHRQ	4
Wang et al. 2016 (25)	Tianjin	955	22.62	case-control study	NRS2002	3, 9, 10, 12, 14, 15, 16, 17, 18, 24	NOS	5
Miu et al. 2018 (26)	Shanghai	142	55.63	cross-sectional study	MQSGA	1, 3, 4, 8, 23	AHRQ	5
Li et al. 2025 (27)	Jiangsu	80	45.00	cross-sectional study	MQSGA	1, 3, 19, 30, 31	AHRQ	4
Zhao et al. 2025 (28)	Beijing	234	42.31	case-control study	MQSGA	1, 2, 3, 4, 19	NOS	7
Zhang et al. 2022 (29)	Liaoning	110	53.64	cross-sectional study	GNRI	1, 8, 19, 27, 28	AHRQ	6
Wang et al. 2016 (30)	Guangdong	114	48.25	case-control study	MQSGA	1, 3, 4, 8, 19	NOS	5
Huang et al. 2023 (31)	Jiangxi	112	35.71	cross-sectional study	MQSGA	1, 3, 4, 14	AHRQ	5
Liu et al. 2024 (32)	Anhui	106	42.45	case-control study	MQSGA	1, 3, 4, 8, 19, 25	NOS	5
Dong et al. 2 019 (33)	Hebei	70	64.29	case-control study	MQSGA	1, 3, 13	NOS	7
Fang et al. 2024 (34)	Hebei	166	49.40	cross-sectional study	MQSGA	1, 2, 3, 4, 5, 6, 7, 8	AHRQ	4
Guo et al. 2025 (35)	Henan	102	29.41	case-control study	MQSGA	6, 10, 14	NOS	5
Yan et al. 2025 (36)	Henan	186	53.76	case-control study	MQSGA	1, 3, 4, 8, 20, 21, 22, 32	NOS	7
Li et al. 2023 (37)	Shandong	85	47.06	case-control study	MQSGA	1, 3, 4	NOS	7
Song et al. 2023 (38)	Guizhou	80	62.50	cross-sectional study	MQSGA	1, 3, 11, 13	AHRQ	5

Assessment tools: MQSGA is the modified quantitative subjective assessment, Q-SGA is the quantitative subjective global assessment for dialysis patients, MIS is the malnutrition-inflammation score, SGA is the subjective global nutritional assessment, GNRI is the geriatric nutritional risk index, and NRS2002 is the nutritional risk screening table. Influencing factors: 1. Age; 2. Body mass index (BMI); 3. Dialysis vintage; 4. Urea clearance index (Kt/v); 5. Serum C-reactive protein (CRP); 6. The awareness rate of nutritional knowledge of kidney disease; 7. Insufficient protein intake; 8. Serum albumin (ALB); 9. Gender; 10. Duration of dialysis per session; 11. Diabetes mellitus; 12. Types of concomitant diseases; 13. Monthly household income; 14. Dialysis frequency; 15. frequency of EPO use; 16. dealing methods; 17. Family function; 18. Social support level; 19. High-sensitivity C-reactive protein (hs-CRP); 20. Depression; 21. Anxiety; 22.(Hb); 23. blood urea nitrogen level (BUN); 24. level of education; 25. live alone; 26. phosphorus (P); 27. parathyroid hormone; 28. Interleukin-6 (IL-6).29. Hand Grip Strength (HGS); 30. Serum creatinine (Scr); 31. Total Cholesterol (TC); 32. Proprotein (PA).

### 3.3 Prevalence of malnutrition in Chinese MHD patients

#### 3.3.1 The overall prevalence of malnutrition

**Figure 2:**
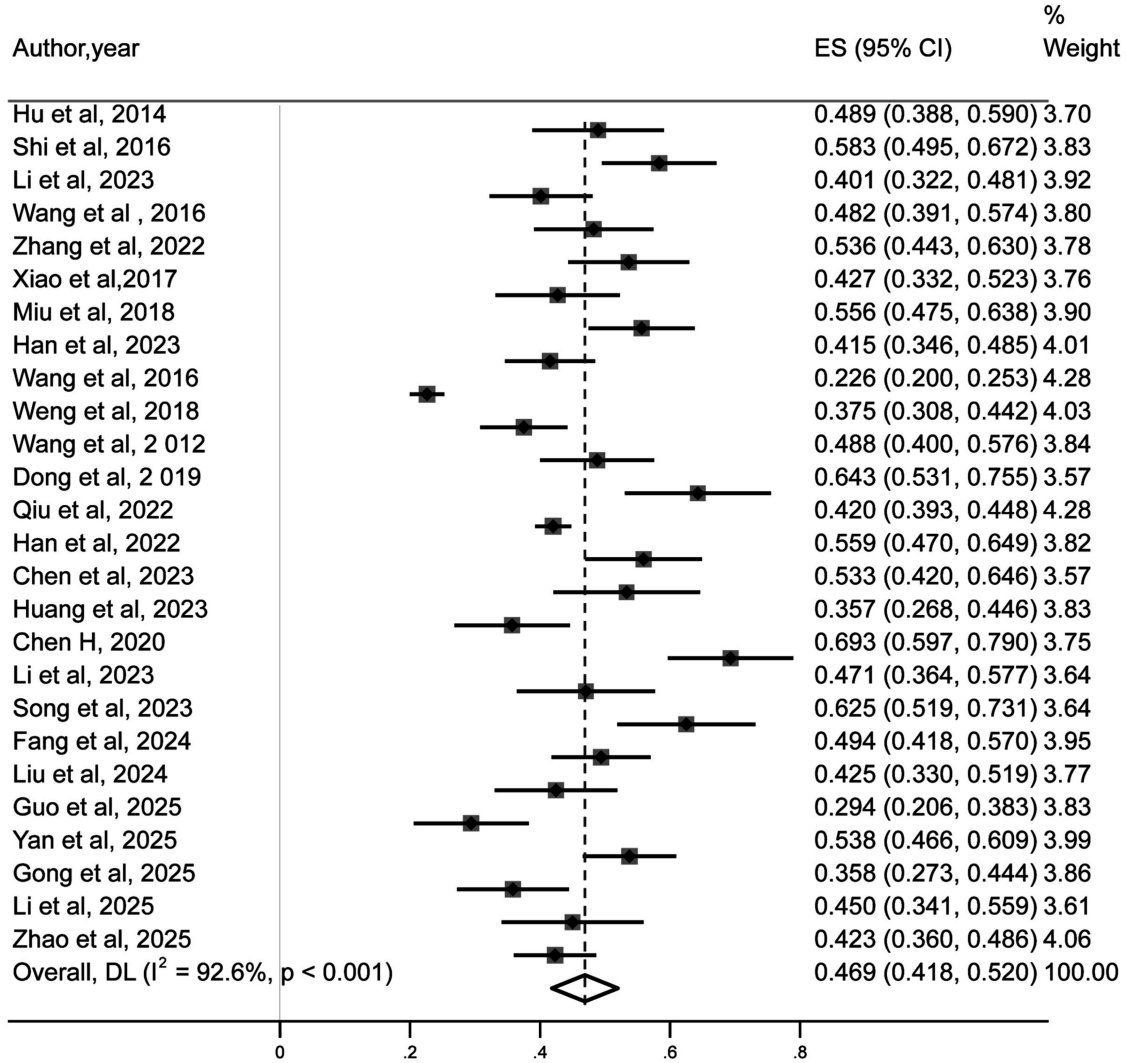
Prevalence of malnutrition in maintenance hemodialysis patients in China.

The analysis of the 26 included studies showed that there was a substantial heterogeneity among the studies (*I*^2^ = 92.6%, *p* < 0.001). A random-effects model was therefore applied, and the results showed that the prevalence of malnutrition in Chinese MHD patients was 46.9% (95% CI: 41.8–52%) as detailed in Figure 2. The assessment tools used to diagnose malnutrition in the original studies of this meta-analysis included the Subjective Global Assessment (SGA), Modified Quantitative Subjective Global Assessment (MQSGA), Quantitative Subjective Global Assessment (Q-SGA), the Malnutrition-Inflammation Score (MIS), the Geriatric Nutritional Risk Index (GNRI), and the Nutritional Risk Screening 2002 (NRS2002).

#### 3.3.2 Subgroup analysis

Subgroup analysis was conducted according to study year, study region, study type, and assessment tool. The studies were stratified by publication year, using 2021 as the cutoff: the prevalence of malnutrition before 2021 (49.4%) was higher than that after 2021 (45.3%); the prevalence of malnutrition was 46.4% (95% CI: 37.5–55.4%) in the case–control study and 47.3% (95% CI: 42.4–52.1%) in the cross-sectional study. Regarding the assessment tools, the prevalence of malnutrition was 69.3% using Q-SGA and 22.6% using NRS2002. The prevalence of malnutrition was 49.0% (95% CI: 43.8–54.2%) in Southern China and 44.7% (95% CI: 36.5–52.9%) in Northern China. The results are shown in Table 3.

**Table 3:** Analysis results of malnutrition prevalence in subgroups

**Subgroup**	**Number of studies**	**Prevalence analysis (I** ^**2**^ **) / (%)**	**Effect model**	**95%CI (%)**	***p-*value**
Study year					
Before 2021	10	96.2%	random	49.4%(37.5%-61.3%)	<0.001
After 2021	16	72%	random	45.3%(41.6%-49%)	<0.001
Study type					
Case - control study	13	95.3%	random	46.4%(37.5%-55.4%)	<0.001
Cross - sectional study	13	82.7%	random	47.3%(42.4 %-52.1%)	<0.001
Assessment tool					
MQSGA	20	76.4%	random-	47.1%(43.4%-50.9%)	<0.001
SGA	2	38.5%	fixed	44.3%(38.9%-49.8%)	0.202
MIS	1	-	-	40.1%(32.2%-48.1%)	<0.001
GNRI	1	-	-	53.6%(44.3%-63%)	<0.001
NRS2002	1	-	-	22.6%(20%-25.3%)	<0.001
Q-SGA	1	-	-	69.3%(59.7%-79%)	<0.001
Study Region					
Southern China	13	83.6%	random-	49.0%(43.8%-54.2%)	<0.001
Northern China	13	95.3%	random	44.7%(36.5%-52.9%)	<0.001

#### 3.3.3 Sensitivity analysis

**Figure 3:**
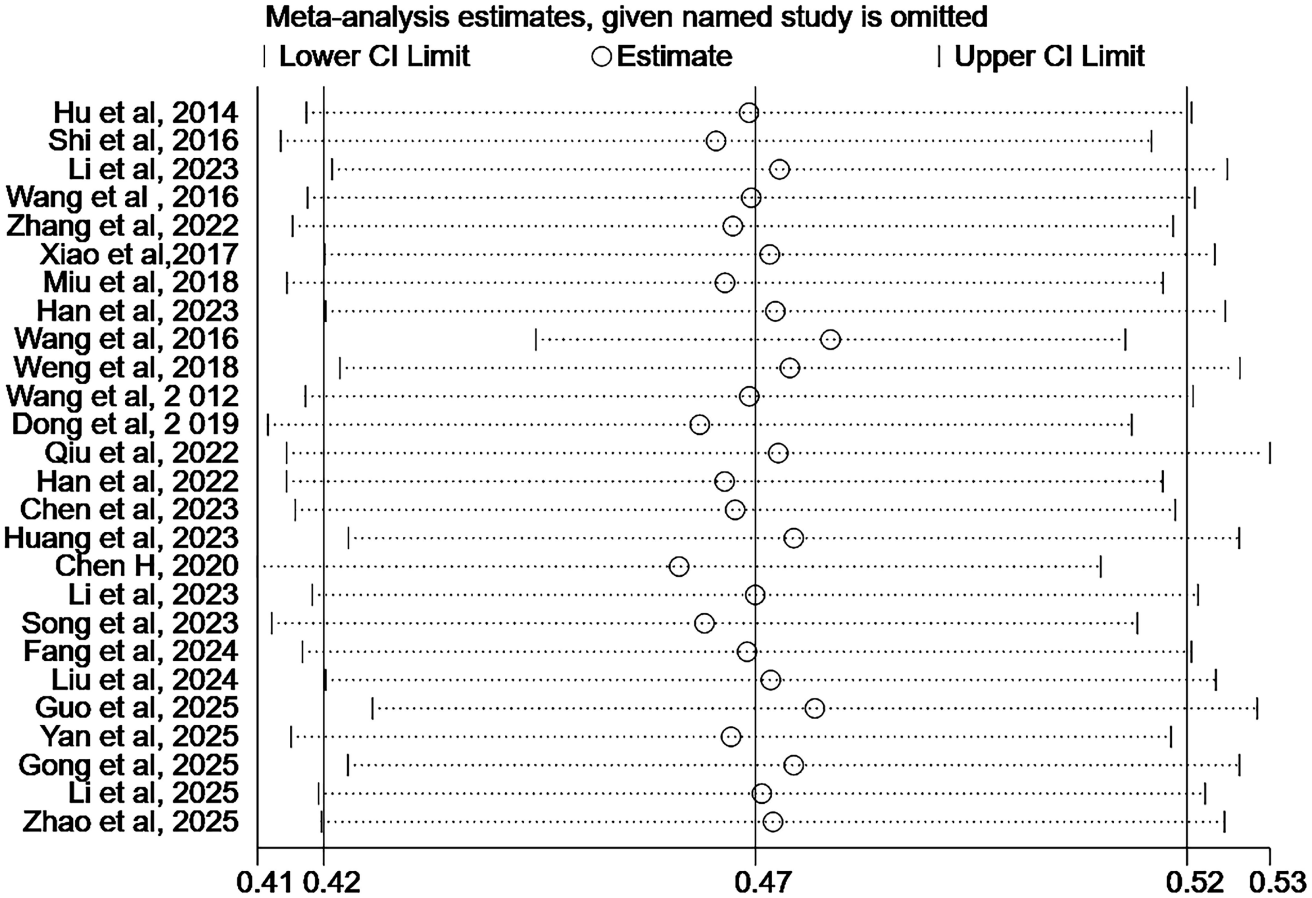
Sensitivity analysis of the prevalence of malnutrition in Chinese MHD patients.

To assess the robustness of the findings, we conducted a sensitivity analysis by excluding individual studies one at a time. The results demonstrated minimal fluctuation in the pooled effect size, thus supporting the stability of the meta-analysis conclusions (Figure 3).

#### 3.3.4 Publication bias test

**Figure 4:**
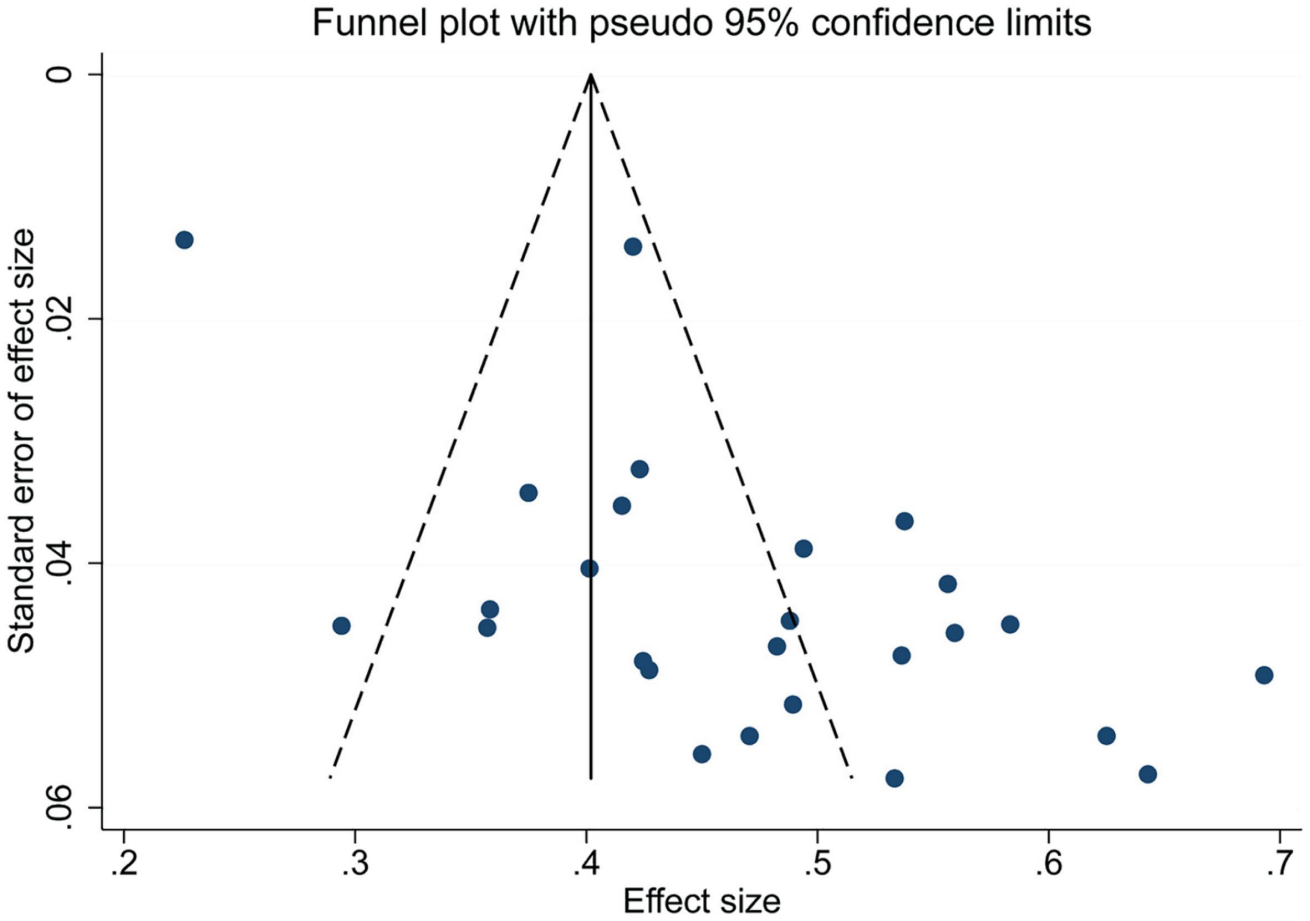
Funnel plot of the prevalence of malnutrition in Chinese MHD patients.

The risk of publication bias was analyzed for the incidence of malnutrition in MHD patients by drawing a funnel plot, and the funnel plot showed asymmetry, suggesting the presence of publication bias, as shown in Figure 4. Egger’s test was significant (*p* < 0.05). Following the trim-and-fill method, it was found that no hypothetical study was included, and the prevalence rate did not change significantly, indicating that the stability of the results was not affected by publication bias.

### 3.4 Influencing factors of malnutrition in Chinese MHD patients

#### 3.4.1 Results of meta-analysis

The meta-analysis revealed multiple significant factors associated with malnutrition in Chinese MHD patients (*p* < 0.05), as detailed in Table 4. These included: age (and age ≥60), BMI, dialysis vintage, Kt/V, CRP, hs-CRP, protein intake, ALB, duration of dialysis per session, monthly household income, frequency of dialysis, frequency of EPO use, depression, and anxiety.

**Table 4:** Influencing factors malnutrition in Chinese MHD patients

**Influencing factors**	**Number of studies**	***I*** ^***2***^ ***(%)***	**Effect model**	**OR value(95%*CI*)**	***p***
Age	20	92.4%	random	1.509(1.364-1.669)	<0.001
Age ≥60	9	81.5%	random	2.850(1.817-4.470)	<0.001
BMI	3	87.6%	random	1.544(0.518-4.602)	<0.001
Dialysis vintage	21	93%	random	2.265(1.813-2.831)	<0.001
Kt/v	15	95.2%	random	2.019(1.243-3.279)	<0.001
CRP	3	69.5%	random	3.013(1.976-4.593)	0.038
The awareness rate of nutritional knowledge of kidney disease	4	0%	fixed	4.495(2.820-7.164)	0.520
protein intake	2	91.4%	random	3.018(0.538 -16.933)	<0.001
ALB	8	93.2%	random	1.115(0.747-1.665)	<0.001
Duration of dialysis per session	3	89.5%	random	1.879(0.515-6.862)	<0.001
Diabetes mellitus	4	0.2%	fixed	2.339(1.882-2.907)	0.391
Monthly household income	3	96.3%	random	1.563(0.644-3.794)	<0.001
Frequency of dialysis	6	91.7%	random	2.100(0.742-5.941)	<0.001
Frequency of EPO use	3	94.4%	random	1.506(0.162-14.022)	<0.001
Family function	2	43.5%	fixed	2.152(1.531-3.024)	0.183
Social support level	2	61.6%	random	2.467(1.052-5.782)	0.106
hs-CRP	5	81.5%	random	2.104(1.393-3.177)	<0.001
Depression	2	96.7%	random	2.671(0.484-14.725)	<0.001
Anxiety	2	96.9%	random	2.531(0.467-13.727)	<0.001

#### 3.4.2 Sensitivity analysis

Sensitivity analysis was performed on studies with the same influencing factors (≥5), and the influencing factors were analyzed by switching between the fixed-effects and random-effects models. The results showed that there was no significant difference in the results except age ≥60 years, BMI, and frequency of EPO use, indicating that the results of meta-analysis were relatively stable, as shown in Table 5.

**Table 5:** Sensitivity analysis of influencing factors of malnutrition in Chinese MHD patients.

Influencing factors	Fixed-effects model		Random-effects model	
	OR value (95%CI)	*p*	OR value (95%CI)	*p*
Age	1.088 (1.069–1.107)	<0.001	1.509 (1.364–1.669)	<0.001
Age ≥60	1.682 (1.456–1.943)	<0.001	2.850 (1.817–4.470)	<0.001
BMI	2.065 (1.675–2.545)	<0.001	1.544 (0.518–4.602)	<0.001
Dialysis vintage	1.054 (1.041–1.068)	<0.001	2.265 (1.813–2.831)	<0.001
Kt/v	2.333 (2.141–2.543)	<0.001	2.019 (1.243–3.279)	<0.001
CRP	2.760 (2.236–3.406)	0.038	3.013 (1.976–4.593)	0.038
The awareness rate of nutritional knowledge of kidney disease	4.495 (2.820–7.164)	0.520	4.495 (2.820–7.164)	0.520
Insufficient protein intake	2.525 (1.543–4.133)	<0.001	3.018 (0.538–16.933)	<0.001
ALB	1.020 (0.955–1.091)	<0.001	1.115 (0.747–1.665)	<0.001
Duration of dialysis per session	0.942 (0.695–1.278)	<0.001	1.879 (0.515–6.862)	<0.001
Diabetes mellitus	2.339 (1.882–2.907)	0.391	2.339 (1.881–2.908)	0.391
Monthly household income	1.041 (0.893–1.213)	<0.001	1.563 (0.644–3.794)	<0.001
Frequency of dialysis	1.705 (1.271–2.286)	<0.001	2.100 (0.742–5.941)	<0.001
Frequency of EPO use	0.644 (0.465–0.890)	<0.001	1.506 (0.162–14.022)	<0.001
Family function	2.152 (1.531–3.024)	0.183	2.148 (1.366–3.378)	0.183
Social support	2.131 (1.351–3.362)	0.106	2.467 (1.052–5.782)	0.106
hs-CRP	1.688 (1.454–1.959)	<0.001	2.104 (1.393–3.177)	<0.001
Depression	1.237 (1.090–1.404)	0.004	2.671 (0.484–14.725)	<0.001
Anxiety	1.131 (1.044–1.226)	<0.001	2.531 (0.467–13.727)	<0.001

### 3.5 Publication bias test

Egger’s test showed that there was no significant publication bias for Kt/V (*p* = 0.535), ALB (*p* = 0.913), frequency of dialysis (*p* = 0.239) and hs-CRP (*p* = 0.171). Significant publication bias was found for age, age ≥60 years, and dialysis vintage (all *p* < 0.05).

## 4 Discussion

### 4.1 Prevalence of malnutrition in Chinese MHD patients

The results of Meta-analysis showed that the prevalence of malnutrition in Chinese MHD patients was 46.9%, which was consistent with the 30.0–66.7% prevalence of malnutrition reported in the guidelines (39). Due to the long-term dialysis treatment, MHD patients often face multiple adverse health factors, including renal failure, poor diet, and malabsorption of nutrients, which may lead to a high prevalence of malnutrition in patients. In addition, subgroup analysis showed that different time periods, regions and study types had certain effects on the prevalence of malnutrition. For example, the prevalence of malnutrition in MHD patients before 2021 and after 2021 was 49.4 and 45.3%, respectively, with a small difference, indicating that this problem has not been effectively alleviated in recent years. From the regional perspective, there was no significant difference in the prevalence of malnutrition between the southern and northern regions (49.0 and 44.7%, respectively), indicating that malnutrition is common in dialysis patients nationwide, and may be related to regional economy, medical resources and patients’ lifestyle.

### 4.2 Age, BMI, dialysis vintage and monthly household income, were the main demographic influencing factors of malnutrition in Chinese MHD patients

Age ≥ 60 years constitutes a significant risk factor for malnutrition in patients undergoing maintenance hemodialysis (MHD). Advancing age is frequently associated with a decline in physiological function and diminished gastric acid secretion, which can impair digestion and nutrient absorption (40). Furthermore, older population often exhibit lower nutritional literacy, have limited access to dietary information, and maintain ingrained eating habits that are resistant to change (41). In addition, Body mass index (BMI) is a widely used measure to assess nutritional status and body composition. An “obesity paradox” has been observed in patients with advanced CKD, especially those on dialysis, in which a relatively high BMI may be associated with a lower risk of death. A higher BMI may indicate a higher nutrient reserve in the body and may reduce the nutrient loss that occurs during dialysis, thereby reducing the risk of malnutrition. However, elevated BMI is not a definitive marker of good nutritional status, since it may also reflect conditions such as sarcopenic obesity, fluid overload, or a chronic inflammatory state. Thus, the nutritional assessment of MHD patients cannot rely on BMI alone, but must be combined with other clinical indicators (42). Therefore, early screening and nutritional intervention, particularly for older and socioeconomically disadvantaged MHD patients, are crucial for improving patient prognosis.

Dialysis vintage demonstrates a positive correlation with malnutrition risk. Study shows that patients dialyzing for more than 4 years exhibit a higher prevalence of malnutrition compared to those with less than 1 year of treatment. This association may be mediated by the exacerbation of inflammatory responses associated with long-term dialysis, which promotes protein catabolism and nutrient depletion, thereby increasing vulnerability to malnutrition (43). Finally, evidence suggests that higher monthly household income is associated with a reduced risk of malnutrition in MHD patients. The substantial financial burden of long-term, regular dialysis may prevent economically disadvantaged patients from affording adequate nutritional supplements. Consequently, nutrient losses sustained during dialysis are not promptly replenished, ultimately contributing to the development of malnutrition (44).

### 4.3 CRP, hs-CRP, protein intake and serum albumin are disease-related factors of malnutrition in Chinese MHD patients

C-reactive protein (CRP) and high-sensitivity CRP (hs-CRP) are key inflammatory cytokines involved in systemic micro-inflammatory responses (45). In MHD patients, this chronic inflammatory state is a central driver of malnutrition. Studies indicate that inflammatory cytokines accelerate protein catabolism and metabolism while activating the IL-6/STAT3 pathway in hepatocytes, which suppresses albumin synthesis and contributes to hypoalbuminemia. Furthermore, these cytokines act on the hypothalamus and gastrointestinal tract to reduce appetite and inhibit gastric acid secretion, thereby impairing nutrient digestion and absorption and further contributing to malnutrition (46). Medical staff should pay attention to the levels of CRP and hs-CRP to identify underlying inflammation, and give corresponding treatment and nursing measures, such as drug therapy and exercise intervention to reduce the inflammatory response and prevent the occurrence of malnutrition.

The Chinese Clinical Practice Guideline for Nutritional Therapy in Chronic Kidney Disease (2021) notes that a low-protein diet is a common therapeutic strategy for MHD patients to alleviate uremic symptoms and slow disease progression (39). However, adequate energy intake must be ensured to meet nutritional demands; otherwise, this dietary restriction may precipitate malnutrition. Serum albumin level is a recognized risk factor for malnutrition in MHD patients, consistent with the findings of Xi et al. (47). As a biomarker of nutritional status, serum albumin reflects the body’s protein reserves. Protein is essential for physiological functions, and a decline in albumin levels reflects protein deficiency, which can lead to metabolic disturbances, a negative nitrogen balance, and ultimately, malnutrition (48). Consequently, it is critical to ensure sufficient protein intake to compensate dialysis-related losses. In clinical practice, dietary guidance should be individualized to balance the necessity of protein restriction with the imperative of adequate nutrition, thereby effectively preventing malnutrition.

### 4.4 Kt/V, duration of dialysis per session, frequency of dialysis and frequency of EPO use are dialysis-related factors of malnutrition in Chinese MHD patients

The urea clearance index (Kt/V), as the core metric for evaluating the adequacy of dialysis, directly reflects the efficiency of waste removal during dialysis, and is an important parameter to evaluate the quality of dialysis and the nutritional status of patients (49). Dialysis adequacy is closely related to the clearance of metabolic wastes in the patient’s body, and insufficient dialysis clearance leads to the accumulation of urotoxins in the body, which can trigger digestive symptoms such as nausea and vomiting. These symptoms, in turn, may impair appetite and reduce nutritional intake. Studies have shown that if the duration of dialysis per session is less than 4 h per time, or the frequency of dialysis is less than 4 times per week, it is often unable to effectively remove metabolic waste in the body, which may lead to insufficient dialysis, thereby affecting the patient’s nutrient absorption and increasing the risk of malnutrition (50). Thus, adequate dialysis is essential for MHD patients. Tailoring the dialysis prescription based on individual patient needs is crucial to ensure adequate solute removal, alleviate clinical symptoms, improve appetite, and thereby help prevent malnutrition.

In addition, Erythropoietin (EPO) is a hormone synthesized primarily by the kidneys, with its key physiological role being the stimulation of erythropoiesis in the bone marrow to maintain red blood cell count and systemic oxygen delivery. In the context of declining renal function, EPO production diminishes, frequently leading to renal anemia in MHD patients (51). Consequently, exogenous EPO is now widely used in the management of anemia associated with MHD. Anemia, a common complication in dialysis patients, shares a close pathophysiological link with malnutrition. Anemia exacerbates tissue hypoxia, impairing cellular energy metabolism and the utilization efficiency of nutrients, thereby worsening pre-existing malnutrition (52). Evidence indicates that EPO administration effectively corrects anemia. Therefore, patients receiving standardized EPO therapy may have a relatively lower risk of developing malnutrition. This association underscores that in addition to ensuring dialysis adequacy, effective management of anemia is equally crucial for improving nutritional status in MHD patients.

### 4.5 Depression and anxiety as psychological influencing factors of malnutrition in Chinese MHD patients

Depression and anxiety are prevalent among MHD patients, often resulting from the high treatment costs and dialysis-related complications, such as fatigue and sleep disturbances (53). These psychological conditions not only manifest as low mood and anhedonia but also significantly impair appetite and eating behavior, leading to reduced or irregular food intake. Chronic loss of appetite and inadequate nutrient intake directly exacerbate malnutrition. Furthermore, depression and anxiety can disrupt gastrointestinal function, altering gastrointestinal motility and secretion, which further impairs nutrient absorption. Collectively, these physiological and psychological pathways significantly worsen the nutritional status of MHD patients (3). Therefore, it is essential for clinical medical staff to pay great attention to the mental health of patients, regularly assess their psychological status, and implement timely, individualized intervention, including psychological counseling, pharmacological treatment and enhanced social support to alleviate symptoms, improve appetite and quality of life, and ultimately reduce the risk of malnutrition.

### 4.6 Limitations

Although this study integrated a substantial amount of data through meta-analysis, there are still several limitations. First, because the types of studies included in the literature were mainly cross-sectional studies and case–control studies, it is difficult to determine the causal relationship. In addition, variability in the quality of the included studies may affect the reliability of the findings. Although sensitivity analysis was performed to assess the influence of individual studies, the conclusions require validation through future prospective research. Second, this study only conducted statistical analysis on some influencing factors. Factors such as urea nitrogen level, educational level, and grip strength could not be further analyzed for the time being due to the limited number of included studies. In the future, high-quality and large-sample prospective cohort studies are needed to address these gaps. Finally, the studies included in this meta-analysis mainly focused on China, and there was considerable heterogeneity in assessment tools and regional focus. Expanding the sample scope to other countries and regions is essential to explore the incidence and influencing factors of malnutrition in MHD patients, thereby providing broader data support for the nutrition management of MHD patients worldwide.

## 5 Conclusion

Malnutrition remains highly prevalent among maintenance hemodialysis (MHD) patients in China. This meta-analysis identified several significant influencing factors, including advanced age, longer dialysis duration, and anxiety or depression. Additionally, several clinical indicators showed a strong positive correlation with malnutrition risk, including lower body mass index (BMI), reduced urea clearance index (Kt/V), elevated serum C-reactive protein (CRP) and high-sensitivity CRP levels, inadequate protein intake, insufficient dialysis frequency or duration per session, and low serum albumin levels. Socioeconomic factors such as lower monthly household income and infrequent use of EPO were also associated with a higher risk of malnutrition. These findings suggest that as these risk factors intensify, the likelihood of malnutrition increases accordingly. Therefore, clinicians should pay close attention to these key determinants, particularly in high-risk groups such as older population, long-term dialysis recipients, those with micro-inflammatory states, individuals with poor nutritional intake, and patients experiencing depression or anxiety. Timely individualized nutritional assessment and tailored intervention strategies are essential to improve nutritional status and overall prognosis in MHD patients.

## Data Availability

The original contributions presented in the study are included in the article/Supplementary material, further inquiries can be directed to the corresponding author.

## Glossary

CKD: Chronic kidney disease
MHD: Maintenance hemodialysis
HD: Hemodialysis
ESRD: End-stage renal disease
RRT: Renal replacement therapy
Mesh: Medical Subject Headings
AHRQ: Agency for Healthcare Research and Quality
NOS: Newcastle Ottawa Scale
SGA: Subjective Global Assessment
MQSGA: Modified Quantitative Subjective Global Assessment
Q-SGA: Quantitative Subjective Global Assessment
MIS: Malnutrition-Inflammation Score
GNRI: Geriatric Nutritional Risk Index
NRS2002: Nutritional Risk Screening 2002
BMI: Body mass index
CRP: C-reactive protein
hs-CRP: high-sensitivity CRP
ALB: Serum albumin
Kt/V: Urea clearance index
EPO: Erythropoietin
