# Supplementary material for: Prevalence and influencing factors of malnutrition in maintenance hemodialysis patients in China: a systematic review
Source: Front Public Health. 2026 Mar 6;14:1771997. doi: 10.3389/fpubh.2026.1771997 (PMC13003832; doi:10.3389/fpubh.2026.1771997)
Supplement: Supplementary file 1 [file Table_1.docx]

Quality assessment

1. Agency for Healthcare Research and Quality，AHRQ

| Item | Yes | No | Unclear |
| --- | --- | --- | --- |
| 1) Define the source of information (survey, record review) |  |  |  |
| 2) List inclusion and exclusion criteria for exposed and unexposed subjects (cases and controls) or refer to previous publications |  |  |  |
| 3) Indicate time period used for identifying patients |  |  |  |
| 4) Indicate whether or not subjects were consecutive if not population-based |  |  |  |
| 5) Indicate if evaluators of subjective components of study were masked to other aspects of the status of the participants |  |  |  |
| 6) Describe any assessments undertaken for quality assurance purposes (e.g., test/retest of primary outcome measurements) |  |  |  |
| 7) Explain any patient exclusions from analysis |  |  |  |
| 8) Describe how confounding was assessed and/or controlled. |  |  |  |
| 9) If applicable, explain how missing data were handled in the analysis |  |  |  |
| 10) Summarize patient response rates and completeness of data collection |  |  |  |
| 11) Clarify what follow-up, if any, was expected and the percentage of patients for which in complete data or follow-up was obtained |  |  |  |

| Study | 1) | 2) | 3) | 4) | 5) | 6) | 7) | 8) | 9) | 10) | 11) | Scores |
| --- | --- | --- | --- | --- | --- | --- | --- | --- | --- | --- | --- | --- |
| Xiao et al, 2017 | Yes | No | Yes | Yes | Unclear | Yes | No | No | No | No | Unclear | 4 |
| Han et al, 2023 | Yes | Yes | Yes | Yes | Unclear | No | No | No | No | No | Unclear | 4 |
| Miu et al , 2018 | Yes | Yes | Yes | Yes | Unclear | No | No | Yes | No | No | Unclear | 5 |
| Zhang et al, 2022 | Yes | Yes | Yes | Yes | Unclear | Yes | No | Yes | No | No | Unclear | 6 |
| Weng et al , 2018 | Yes | No | Yes | Yes | Unclear | Yes | No | No | No | No | Unclear | 4 |
| Chen H, 2020 | Yes | Yes | No | Yes | Unclear | Yes | No | Yes | No | No | Unclear | 5 |
| Qiu et al, 2022 | Yes | Yes | Yes | Yes | Unclear | Yes | No | No | No | No | Unclear | 4 |
| Wang et al, 2 012 | Yes | Yes | Yes | Yes | Unclear | Yes | No | No | No | No | Unclear | 5 |
| Huang et al, 2023 | Yes | Yes | Yes | Yes | Unclear | No | No | Yes | No | No | Unclear | 5 |
| Song et al, 2023 | Yes | Yes | Yes | Yes | Unclear | No | No | Yes | No | No | Unclear | 5 |
| Fang et al, 2024 | Yes | Yes | Yes | Yes | Unclear | No | No | No | No | No | Unclear | 4 |
| Gong et al, 2025 | Yes | Yes | No | Yes | Unclear | Yes | No | No | No | No | No | 4 |
| Li et al, 2025 | Yes | Yes | Yes | Yes | Unclear | No | No | No | No | No | No | 4 |

2. Newcastle-Ottawa Scale (NOS)

| Section | Item | Evaluation criteria |
| --- | --- | --- |
| Selection | 1) Is the case definition adequate? | a) yes, with independent validation*；  b) yes, eg record linkage or based on self reports; c) no description |
|  | 2) Representativeness of the cases | a) consecutive or obviously representative series of cases*;  b) potential for selection biases or not stated |
|  | 3) Selection of Controls | a) community controls*; b) hospital controls;  c) no description |
|  | 4) Definition of Controls | a) no history of disease (endpoint)*;  b) no description of source |
| Comparability | Comparability of cases and controls on the basis of the design or analysis | a) study controls for ___ (Select the most important factor) *;  b) study controls for any additional factor* (This criteria could be modified to indicate specific control for a second important factor.) |
| Exposure | 1) Ascertainment of exposure | a) secure record (eg surgical records) *;  b) structured interview where blind to case/control status*;  c) interview not blinded to case/control status;  d) written self report or medical record only;  e) no description |
|  | 2) Same method of ascertainment for cases and controls | a) yes*; b) no |
|  | 3) Non-Response rate | a) same rate for both groups*;  b) non respondents described;  c) rate different and no designation |
| Note: A study can be awarded a maximum of one star for each numbered item within the Selection and Exposure categories. A maximum of two stars can be given for Comparability. | | |

|  | Selection | | | | Comparability | Exposure | | |  |
| --- | --- | --- | --- | --- | --- | --- | --- | --- | --- |
| Study | case definition adequate | Representativeness of the cases | Selection of Controls | Definition of Controls | Comparability of cases and controls on the basis of the design or analysis | Ascertainment of exposure | Same method of ascertainment for cases and controls | Non-Response rate | Scores |
| Hu et al, 2014 | 1 | 1 | 0 | 1 | 2 | 1 | 1 | 0 | 7 |
| Shi et al, 2016 | 1 | 1 | 0 | 1 | 1 | 1 | 1 | 0 | 6 |
| Li et al, 2023 | 1 | 1 | 0 | 1 | 2 | 1 | 1 | 0 | 7 |
| Chen et al, 2023 | 1 | 1 | 0 | 1 | 2 | 1 | 1 | 0 | 7 |
| Li et al, 2023 | 1 | 1 | 0 | 1 | 0 | 1 | 1 | 0 | 5 |
| Wang et al, 2016 | 1 | 1 | 0 | 1 | 0 | 1 | 1 | 0 | 5 |
| Wang Q et al, 2016 | 1 | 1 | 0 | 1 | 0 | 1 | 1 | 0 | 5 |
| Han et al, 2023 | 1 | 1 | 0 | 1 | 1 | 1 | 1 | 0 | 6 |
| Dong et al, 2 019 | 1 | 1 | 0 | 1 | 2 | 1 | 1 | 0 | 7 |
| Liu et al, 2024 | 1 | 1 | 0 | 1 | 0 | 1 | 1 | 0 | 5 |
| Guo et al, 2025 | 1 | 1 | 0 | 1 | 0 | 1 | 1 | 0 | 5 |
| Yan et al, 2025 | 1 | 1 | 0 | 1 | 2 | 1 | 1 | 0 | 7 |
| Zhao et al, 2025 | 1 | 1 | 0 | 1 | 2 | 1 | 1 | 0 | 7 |
